# Outcomes of home health care and telephone home visit by pharmacist in type 2 diabetes patients on hospital readmission: a case study at Bangplama Hospital, Suphanburi Province, Thailand

**DOI:** 10.1186/1471-2458-12-S2-A20

**Published:** 2012-11-27

**Authors:** Nilawan Upakdee, Suwicha Mankongdee

**Affiliations:** 1 Faculty of Pharmaceutical Sciences, Naresuan University, Phitsanulok, Thailand; 2 Pharmacy Department, Bangplama Hospital, Suphanburi, Thailand

## Background

The objective of this study was to compare outcomes of home health care and telephone home visit by pharmacist in type 2 diabetes patients on hospital readmission at Bangplama hospital, Suphanburi Province.

## Materials and methods

Inclusion criteria was admitted patients with first diagnosed conditions with low or high blood sugar level. This study was done during December 2010 to February 2011. This study was true experimental design with randomized block that is similar in co-morbidites and age range. Fourteen patients were classified into two groups, each group composed of 7 cases. Control group received home health care and study group received telephone home visit. Primary outcome was readmission within 28 days. Economic outcome was charge of care savings from preventing hospital readmission.

## Results

The results showed that control group had none of patient readmission, while 14.3% of study group was readmitted. Clinical outcomes were mean fasting blood sugar (FBS) and blood pressure (BP) first time at discharge and second time within 28 days. In control group, average mean FBS were 175.29 mg/dl and 154.29 mg/dl, respectively. In the study group, average mean FBS were 120.57 mg/dl and 120.85 mg/dl, respectively. Systolic blood pressure in the control group was decreased at 129.14 mmHg and 117.85 mmHg, respectively. While in the study group were almost equal to 137.00 mmHg and 135.85 mmHg, respectively. Diastolic blood pressure in the control group was increased at 79.57 mmHg and 82.145 mmHg, respectively. In the study group were increased at 76.85 mmHg and 81.28 mmHg, respectively. Direct medical care charge from prevent hospital readmission in control and study group were 49,905 Baht and 40,108 Baht, respectively.

## Conclusions

The results of this study show that both home health care and telephone home visit by pharmacist in diabetes patients were beneficial for preventing readmission.

